# Effects of Ego-Depletion and State Anxiety on Performance Changes in Dart-Throwing Tasks: A Latent Curve Model Approach Reporting Trial Data for Human Participants

**DOI:** 10.3389/fpsyg.2019.02027

**Published:** 2019-09-04

**Authors:** Jonghyun Yang, Kiwon Park, Myoungjin Shin

**Affiliations:** ^1^Department of Physical Education, Incheon National University, Incheon, South Korea; ^2^Sport Science Institute, Incheon National University, Incheon, South Korea; ^3^Department of Mechatronics Engineering, Incheon National University, Incheon, South Korea; ^4^Research Institute for Engineering and Technology, Incheon National University, Incheon, South Korea; ^5^Research Institute for Basic Sciences, Soonchunhyang University, Asan, South Korea

**Keywords:** ego depletion, perceptual-motor task, performance change, state anxiety, dart-throwing

## Abstract

This study examined the effects ego depletion (ED) and state anxiety (SA)have on performance changes in dart-throwing, a perceptual-motor task, using a Latent Curve Model (LCM). Twenty-two men, who had never played darts before, were recruited and randomly assigned to two counterbalanced groups: Group A was exposed first to ED and then to non-ED (NED), and Group B was exposed first to NED and then to ED. We found that the number of trials had a non-linear effect on darts performance, which improved as the number of trials increased before declining again; that is, mean radial error decreased first and then increased. Therefore, motor performance was sustained in the form of a quadratic curve as the number of task executions increased. In general, the higher the degree of ED and SA, the greater the negative effect on performance. However, this phenomenon was observed only in the early-phase trials, and the interacting influence of ED and SA appeared in the late-phase trials. Thus, this study demonstrated that ED and SA have direct effects on performance curves in early-phase trials only, and that their interacting influence appears in late-phase trials.

## Introduction

Self-control is the capacity to change one’s response based on own ideals, values, morals, social expectations, or long-term goals (Baumeister et al., 2007). Without self-control, it could be impossible to achieve goal-oriented behavior or long-term goals (Loewenstein, 1996). Baumeister et al. (1994) proposed the strength model that addresses the working principle of self-control and the failure of control based on ego depletion (ED; Baumeister et al., 2007).

According to the strength model, the strength necessary to perform any action is a finite self-control resource; however, when in a state of ED, it is difficult to execute actions and behaviors that require self-control resources, as the self-control resource is depleted (Baumeister et al., 1994, 1998). The self-control resource can be considered as being similar to the energy necessary for muscle contraction; although the energy necessary for muscle contraction is finite, it becomes available again after a certain period of time (Baumeister et al., 1994, 1998). In other words, if the upper limit is reached, which leads to complete depletion of the self-control resource, a state of ED commences, which makes it impossible to use the self-control resource; however, the resource recovers over time, and is available again at a later time point. Therefore, ED refers to a state in which the self does not have all the resources that it normally has and renders the self temporarily less able and less willing to function normally or optimally (Baumeister and Vohs, 2007).

Self-control strength in sports contexts has been shown to negatively impact performance by weakening impulse regulation (Englert, 2016). Englert et al. (2015b) investigated the effect of ED on the frequency of false starts in starting blocks for female soccer players with no previous experience of track and field events. The results from this study showed that the frequency of false start was higher in the ED group than the non-ED group because the ED group could not suppress the urge to start faster.

And previous studies of the correlation between motor performance and ED can be divided into two categories: those that have examined persistence tasks and those that have examined perceptual-motor tasks. A commonly used persistence task in such studies is squeezing an isometric handgrip (Muraven et al., 1998; Ciarocco et al., 2001; Finkel et al., 2006). Squeezing such a handgrip involves an experiment subject clenching an instrument (typically a hand dynamometer) in their hand for as long as possible, which requires a certain level of hand-muscle strength, depending on the experiment parameters. Greater pain is perceived as the duration of the hand-gripping increases, and here self-control strength is necessary to suppress the desire to end the task (Muraven et al., 1998). Studies that have examined the correlation between ED and isometric-handgrip tasks (Bray et al., 2008, 2011) have found that ED decreases performance in such tasks; further, ED manipulation has also been reported to reduce performance in other persistence-based tasks such as cycling (Wagstaff, 2014; Englert and Wolff, 2015) and press-ups/sit-ups (Dorris et al., 2012).

Meanwhile, a perceptual-motor task is an attention-demanding motor task that involves selectively focusing on task-relevant stimuli and disregarding information that has no or low relevance to the task at hand (Abernethy et al., 2007; Wilson et al., 2009; Englert et al., 2015c). In perceptual-motor tasks, anxiety or pressure to perform tends to impair task performance by interfering with the ability to use performance-related information. This phenomenon has been termed “choking under pressure” (Baumeister, 1984). Thus, performance of a perceptual-motor task is expected to decrease (i.e., worsen) when executed in a high-anxiety situation (Behan and Wilson, 2008; Wilson et al., 2009); however, there are also reports of contrary findings (Craft et al., 2003; Woodman and Hardy, 2003). Englert and Bertrams (2012) and Englert et al. (2015a) attributed these contradictory research findings to individual differences relating to level of self-control strength. In their studies, ED groups showed a higher negative correlation between anxiety and performance during perceptual-motor tasks (e.g., dart-throwing, making basketball free throws) than did non-ED groups (NED groups). Moreover, the phenomenon of reduced gaze fixation on task-relevant information in high-anxiety situations was observed more frequently in their ED groups than in their NED groups. This implies that, even in high-anxiety situations, anxiety affects performance to a lesser degree if sufficient self-control strength is available. In contrast, if self-control strength is completely depleted, the negative effect of anxiety on performance is reinforced.

Rapid adaptation to changing environments is a form of motor ability (Stöckel and Fries, 2013). According to the theory of generalized motor programs, rapid motor adaptation to a variety of environmental changes by perceiving changes in environments and adapting to them through internal representation of their movement is an important factor in motor ability. The veracity of this theory has been supported by studies on basketball players, where motor performance was impaired by manipulation of the basket’s height (De Oliveira et al., 2009) and the ball’s weight (Breslin et al., 2010), but tended to gradually improve with repeated trials, which was a result of adaptation to task-related context information (De Oliveira et al., 2009).

However, previous studies investigating the correlations of ED and anxiety with motor performance in perceptual-motor tasks (e.g., Englert and Bertrams, 2012; Englert et al., 2015c) have based their analyses on mean scores in overall performance outcomes and, thus, are compromised by the limitations of evaluating individual motor ability through results of motor performance alone, without considering changes in performance (or adaptation) as the number of trials increases. To overcome this limitation, in the present study we examined the effects of ED and anxiety on motor performance. Through this approach, we sought to verify the effect the interaction between ED and anxiety has on changes in performance as the number of trials increases, using dart-throwing as the perceptual-motor task for the experiment. To this end, we formulated the following hypotheses:

Hypothesis 1: The higher the number of trials, the better the performance.

Hypothesis 2: The higher the participant’s perceived ED and state anxiety (SA), the lower the extent of change in his/her performance.

Hypothesis 3: Changes in performance are influenced by the interaction between the participant’s perceived ED and his/her SA.

## Materials and Methods

### Participants

We recruited 22 men in their early twenties who were native speakers of Korean, had never played darts before, and who had no impairments in their upper extremities. These participants were randomly assigned to one of two counterbalanced groups: Group A (*n* = 11, 22.9 ± 1.4 years., 177.5 ± 5.9 cm, 71.1 ± 5.6 kg), which was first exposed to ED, and then to NED (ED-NED group), and Group B (*n* = 11, 23.0 ± 1.2 years., 176.9 ± 3.2 cm. 72.5 ± 5.3 kg), which was initially exposed to NED, and then to ED (NED-ED group). The study was conducted in accordance with the Declaration of Helsinki, and the protocol was approved by the Ethics Committee. All participants provided written informed consent to participate, after being provided with sufficient explanations regarding the purpose, content, and method of the study.

### Apparatus and Manipulation Task

**FIGURE 1:**
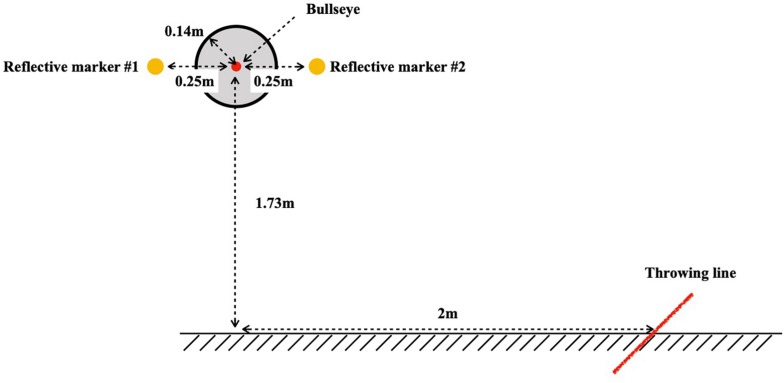
Layout of the dartboard and throwing line.

**FIGURE 2:**
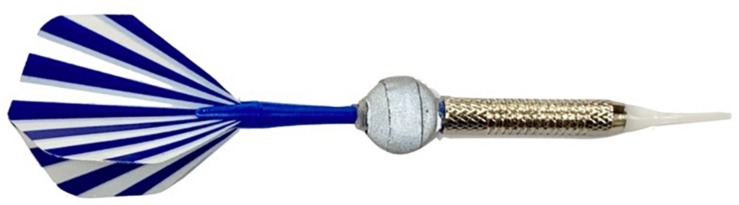
Soft dart with reflective marker.

We computed each participant’s MRE using eight infrared cameras (OptiTrack Prime 17, United States) and an electronic dartboard (WJ100, China). As illustrated in Figure 1, the dartboard was mounted at a height of 1.73 m (from the floor to the center of the bullseye), which is the standard height for a darts competition, and the throwing distance was set at 2 m (from the ground point directly under the center of the bullseye to the throwing line). For measurement, the center of the dartboard was determined by attaching a reflective marker on each side of the dartboard, 25 cm from the center of the bullseye, and the position of the dart was monitored using a reflective marker mounted between the barrel and the shaft of the dart (length: 14 cm), as shown in Figure 2. These reflective markers were then detected using the infrared cameras. If a dart fell out of the dartboard after hitting it, the score for the point closest to the place hit was taken as the performance.

Generally, in the case of steel darts, the score is not recognized when the darts fall out of the dartboard. As soft darts were used in this experiment, sensor determination was prioritized. As shown in Figure 2, the pin of soft dart was made of plastic, unlike steel darts, and the darts could have fallen out of the dartboard. In the case of the actual game, it follows the judgment of the sensor, but in this experiment, it was measured more accurately using the three-dimensional coordinate value.

For ED manipulation, we used a writing task, which we modified from a similar task developed by Bertrams et al. (2010). Bertrams et al.’s original writing task was designed to induce a state of ED by instructing participants to transcribe a given English text, but to omit certain common letters of the alphabet. However, since the participants in this study were not native speakers of English, we used Korean texts instead of English; this was to avoid ED during NED manipulation, as transcribing in a non-native language could inadvertently induce ED, regardless of the parameters of the task. Thus, during the ED manipulation, our participants were instructed, after reading an article that concerned a social issue, to transcribe the article without using specific vowels (“
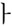
” and “
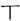
”) and consonants (“
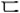
” and “
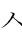
”). Meanwhile, during the NED manipulation, the participants were instructed to transcribe an article about a famous sportsman without omitting any specific vowels or consonants, which should result in minimal ED.

In previous studies, SA manipulation was performed through various methods [e.g., listening to audio recordings of anxiety experienced by athletes during free-throw situations (Englert et al., 2015a), or throwing a dart at a target suspended 7-m from ground (Englert et al., 2015c)]. In the present study, we manipulated SA by inducing competition and evaluative concerns based on Englert and Bertrams (2012)’s study. All participants were notified that poor scores in the dart-throwing task could adversely influence the total experiment results, and that the top three scorers would win additional rewards. Thus, SA was induced by introducing additional pressure to perform in the experiment, as well as a competition situation.

### Experimental Procedures

The participants were taught how to throw a dart from the throwing line, were shown three demonstration shots, and were allowed six trial shots. Then, after responding to items concerning demographics (age, height, and weight) and a dart-related self-efficacy questionnaire, the participants performed a total of 15 shots (five sets of three shots), which served as their baseline performance [MRE (pre)] in the later measurements.

**FIGURE 3:**
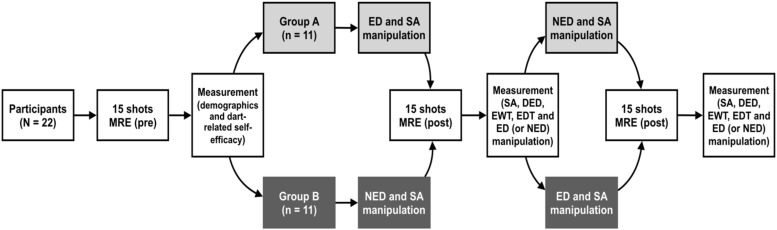
Flow chart of the experiment procedure. MRE, mean radial error; ED, ego depletion; NED, non-ego depletion; SA, state anxiety; DED, degree of ego depletion; EWT, effort invested in the writing task; EDT, effort invested in the dart-throwing task.

Group A then performed the ED and SA manipulation task, and the first dart-throwing task (a total of 15 shots, five sets of three shots), and then responded to questionnaires regarding SA, degree of ego depletion (DED), effort invested in the writing task (EWT), effort invested in the dart-throwing task (EDT), and ED manipulation^1^ for the first dart-throwing task. Next, they underwent the NED task and the second dart-throwing task (a total of 15 shots, five sets of three shots), and responded to the questionnaires regarding SA, DED, EWT, EDT, and NED manipulation for the second dart-throwing task. Group B underwent the same procedure, with the exception of executing the NED task first, counterbalancing the order of the experiments (Group A: ED-NED; Group B: NED-ED). In this study, SA manipulation was performed before the dart task, and SA was measured after the dart task execution was completed. The flow chart of the experiment procedure is shown in Figure 3.

In Table 1, MRE (pre) represents the baseline darts performance (measured using the initial 15 shots), and MRE (post 1) to MRE (post 5) represent the darts performance for the five sets of three shots, respectively, executed after each ED or NED. Changes in darts performance can be tracked by comparing the scores of the five sets of shots from MRE (post 1) to MRE (post 5) with those of MRE (pre). The mean score of MRE decreased continuously from MRE (pre) to MRE (post 4) (early-phase trials), while the score increased from MRE (post 5) (late-phase trials). Therefore, the participants’ dart throwing performance showed non-linear patterns as the number of trials increased.

**TABLE 1:** Descriptive statistics and correlation analysis.

	**1**	**2**	**3**	**4**	**5**	**6**	**7**	**8**
1. MRE (pre)	–							
2. MRE (post 1)	0.41^∗∗^	–						
3. MRE (post 2)	0.07	0.11	–					
4. MRE (post 3)	0.27	0.19	0.14	–				
5. MRE (post 4)	0.14	0.20	0.28	0.33^∗^	–			
6. MRE (post 5)	0.41^∗∗^	–0.04	–0.07	0.13	0.31^∗^	–		
7. SA	–0.16	0.25	–0.04	0.17	–0.02	–0.27	–	
8. DED	–0.18	–0.01	–0.08	0.26	0.02	–0.06	0.25	–
Mean (SD)	87.40 (24.82)	84.62 (27.97)	76.42 (31.36)	72.19 (22.01)	81.03 (28.58)	75.53 (31.97)	3.57 (1.95)	5.38 (1.85)
Skewness	1.72	1.12	0.29	0.41	0.69	0.70	0.85	–1.15
Kurtosis	1.76	1.21	0.14	–0.07	0.77	0.40	0.67	0.30

*MRE, mean radial error; SA, state anxiety; DED, degree of ego depletion. ^∗^*p* < 0.05, ^∗∗^*p* < 0.01.*

Diallo et al. (2014) reported that statistical power is strongly influenced by measurement points and sample size in non-linear latent models and the power at six measurement points was 0.959 and it was 0.80 when calculated with 50 cases. In this study, there were six measurement points: MRE (pre), MRE (post 1), MRE (post 2), MRE (post 3), MRE (post 4), and MRE (post 5) and the total number of cases counterbalanced was 44, which is close to 50. Thus, the statistical power of 0.8 was achieved.

### Measurement Tools

#### State Anxiety

We measured each participant’s SA while executing the task using the Mental Readiness Form 3 (Krane, 1994), after modifying it to suit the purpose of this study. This tool comprises four items, which are rated using an 11-point Likert scale:

1. “I felt pressure to avoid failure in the dart-throwing task” (1 = not at all; 11 = extremely).
2. “I felt my body become tense while performing the dart-throwing task” (1 = not at all; 11 = extremely).
3. “I felt capable of adequately performing the dart-throwing task” (1 = extremely, 11 = not at all).
4. “I felt worried while performing the dart-throwing task” (1 = not at all, 11 = extremely).

#### Degree of Ego Depletion

As Baumeister (2014) insists that fatigue is more likely when exposed to ED, the degree of ED in this study was measured to determine the degree of psychological fatigue. We measured each participant’s level of ED using a one-item questionnaire that has been used in previous research (Englert and Bertrams, 2012; Englert et al., 2015c), after modifying it to suit the purpose of this study:

“How much mental fatigue did you experience after the writing task?” (1 = none at all, 7 = a great deal).

#### Darts Performance

Darts performance was evaluated based on dart-throwing accuracy; the closer the dart landed to the bullseye (Figure 1), the higher the performance. As a measure of dart-throwing accuracy, we used the mean radial error (MRE)^2^, expressed in the formula below:

$$M\,R\,E=\frac{1}{n}\,\sum_{i=1}^{n}\sqrt{x_{i}^{2}+y_{i}^{2}}$$

*x*_*i*_, *y*_*i*_: the coordinates of the point at the *i*-th trial, *n*: the total number of trials, the unit of MRE measurement was mm.

#### Control Variables

As per the social cognitive theory of Bandura (1997, 2006), self-efficacy is one of the most important psychological variables predicting human behavior and performance, and Englert (2016) insists that it is important to consider other psychological processes when dealing with ED and performance in the study. Therefore, self-efficacy in this study was used as a control variable. Using the Guide for Constructing Self-efficacy Scales (Bandura, 2006) as a foundation, we developed a three-item questionnaire to measure self-efficacy in throwing darts:

1. “I believe I can throw darts better than can the other participants.”
2. “I believe that, with practice, I can improve my darts performance to be better than that of the other participants.”
3. “I think that my final score will be one of the highest among all participants in the experiment.”

The participants were instructed to indicate their degree of conviction in their ability by using a 100-point scale (0–100%) to score each item.

Next, the degree of effort invested in the writing task was measured using a one-item questionnaire, which was rated using a seven-point Likert scale (1 = not at all, 7 = a great deal):

“How hard did you try to finish the writing task?”

Then, the degree of effort invested in the dart-throwing task was measured using a two-item questionnaire, with both questions rated using an 11-point Likert scale:

1. “I to succeed in the dart-throwing task” (1 = made no effort at all, 11 = tried to the best of my ability).
2. “I to succeed in the dart-throwing task” (1 = did not concentrate at all, 11 = concentrated to the best of my ability).

ED task is usually manipulated to reduce the expectation of success for the task (Englert and Bertrams, 2012) because of the suppression of the routine of the self, and based on the items used in the previous study (Englert et al., 2015c). To measure the extent of ED manipulation, we developed a two-item questionnaire, rated using a seven-point Likert scale, by modifying items used in previous studies (Englert and Bertrams, 2012; Englert et al., 2015c):

1. “How hard did you try to overcome your usual writing routine (overall writing habits such as writing speed and concentration) while executing the writing task?” (1 = not at all, 7 = a great deal).
2. “Do you think you have successfully executed the writing task, including providing correct spelling and spacing?” (1 = not at all, 7 = very much so).

### Data Analysis

The Latent Curve Model (LCM), which is a method of analyzing trends in cross-sectional data within the framework of structural equation modeling, lends itself well to examining performance changes over a varying number of trials. There are two steps in the LCM. In the first step, the slope of the MRE and the initial value of the unconditional LCM are estimated. If the variance of the estimated MRE slope and the initial value is statistically significant, conditional LCM analysis is performed at the second stage. In this study, we investigated whether the changes in MRE due to the number of trials are linear and quadratic curves (non-linear change models) using the fitness indices of TLI, CFI, and RMSEA. The effects of ED and SA on darts performance were examined using the LCM available in AMOS 18.0. Parameter estimation was performed using the maximum likelihood method, and an alpha (*p*-value) of 0.05 was set as the cutoff for statistical significance.

## Results

### Descriptive Statistics

Table 1 shows the non-linear relationship between the MRE and the number of trials, whereby the MRE decreases with the increase in the number of trials, before increasing again. Given the low correlation coefficients between all input variable pairs, the problem of multicollinearity was negligible, and the skewness and kurtosis values (± 2 or lower) are indicative of normal distribution.

### Randomization and Manipulation Check

The independent *t*-test was performed with the participants randomized into two counterbalanced groups: Group A (*n* = 11) and Group B (*n* = 11). The effectiveness of the randomization was checked by considering both self-efficacy in darts and age. Consequently, we found no statistically significant differences between Group A and Group B regarding self-efficacy (*t*(20) = 1.20, *p* = 0.243), age (*t*(20) = 0.48, *p* = 0.632), and MRE (pre) (*t*(20) = 0.07, *p* = 0.944); thus, we determined the randomization to be adequate.

The paired *t*-test on the ED-manipulation check showed that the ED measurement point (*M* = 4.70, *SD* = 0.72) scored higher than did the NED measurement point (*M* = 4.00, *SD* = 1.33) in the manipulation-check questions, with the difference being statistically significant (*t*(21) = 2.33, *p* < 0.05). Further, the ED measurement point (*M* = 6.5, *SD* = 0.80) also scored higher than did the NED measurement point (*M* = 4.27, *SD* = 1.96) in regard to degree of ED, also to a statistically significant level (*t*(21) = 5.00, *p* < 0.001).

In line with our assumptions, there was a significant difference in ED based on the number of transcribed characters (*t*(21) = 20.39, *p* < 0.001), as depleted participants (*M* = 242.09, *SD* = 27.47) transcribed a smaller number of characters than non-depleted participants (*M* = 444.82, *SD* = 32.31). The percentage of errors was significantly higher in the depleted state (*M* = 6.60, *SD* = 1.80) than in the non-depleted state (*M* = 0.47, *SD* = 0.07), *t*(21) = 16.01, *p* < 0.001. No statistically significant intergroup differences were observed regarding effort invested in the writing task (*t*(21) = 1.91, *p* = 0.070) or in the dart-throwing task (*t*(21) = −0.20, *p* = 0.845). From these results, we verified that the writing task, in which specific vowels and consonants were omitted, induced temporary ED, and that the participants put the same level of effort into the writing and dart-throwing tasks in both the ED and NED conditions.

In Table 1, the normal distribution of SA is indicative of the individual differences among the participants regarding the level of SA, which implies that the pressure imposed on the participants concerning the experiment and competition situation adequately served to meet the normal distribution assumption.

### Linear Change in Darts Performance (Unconditional Model)

A linear LCM was used to investigate whether the MRE decreases linearly with increase in the number of trials. The slope path coefficients were assigned to change linearly to 0, 1, 2, 3, 4, and 5 at regular intervals. The results showed that it was statistically significant (χ^2^ (16) = 12.67, *p* < 0.05) and the fit values were unacceptable (TLI = 0.260, CFI = 0.211, RMSEA = 0.331). Therefore, the conditional model reflecting the linear decreasing tendency of the MRE was statistically irrelevant.

### Non-linear Change in Darts Performance (Unconditional Model)

Using LCM analysis, we examined the non-linear change in MRE, exhibited as a quadratic curve in Table 1, in which MRE decreased as the number of trials increased, before increasing again. As a result, no statistically significant differences were observed in the unconditional model, as shown in Table 2 (χ^2^(12) = 12.67; *p* = 0.393), demonstrating that there were no statistically significant differences between the LCM selected as the quadratic curve and the raw data. As the fit values (TLI = 0.963, CFI = 0.971, RMSEA = 0.036) were found to be superior as well, we concluded that the statistically non-linear conditional model was appropriate.

**TABLE 2:** The result of LCM.

		**Unconditional Model**	**Conditional Model**
Intercept	Mean	87.49^∗∗∗^	112.56^∗∗∗^
	Variance	555.04^∗∗^	552.26^∗∗^
Linear Slope	Mean	−6.76^∗^	–61.29^∗∗^
	Variance	339.66^∗∗^	233.29^∗^
Non-linear Slope	Mean	0.89	12.78^∗∗^
	Variance	15.70^∗∗^	10.70^∗^
Model Fit	χ^2^(df)	12.67 (12)	21.41 (21)
	TLI	0.963	0.996
	CFI	0.971	0.997
	RMSEA	0.036	0.021

*TLI, Tucker-Lewis index; CFI, comparative fit index; RMSEA, root mean square of approximation. ^∗^*p* < 0.05, ^∗∗^*p* < 0.01, ^∗∗∗^*p* < 0.001.*

The intercept was 87.49, very similar to the MRE (pre) mean shown in Table 1 (87.40). However, whereas the linear slope was statistically significant (−6.76; *p* < 0.05), the non-linear slope showed no statistical significance (0.89; *p* = 0.215). This indicates the existence of a non-linear relationship, in which the decreasing trend in MRE observed in the early-phase trials was not maintained from the fourth set of three shots (post 4) onward. Error variance was statistically significant in relation to intercept, linear slope, and non-linear slope. Since this suggests the statistical feasibility that this variance can be explained by other variables, we performed a conditional model analysis.

### Effects of Ego Depletion Dependent Upon State Anxiety on Sustaining Change in Darts Performance (Conditional Model)

**FIGURE 4:**
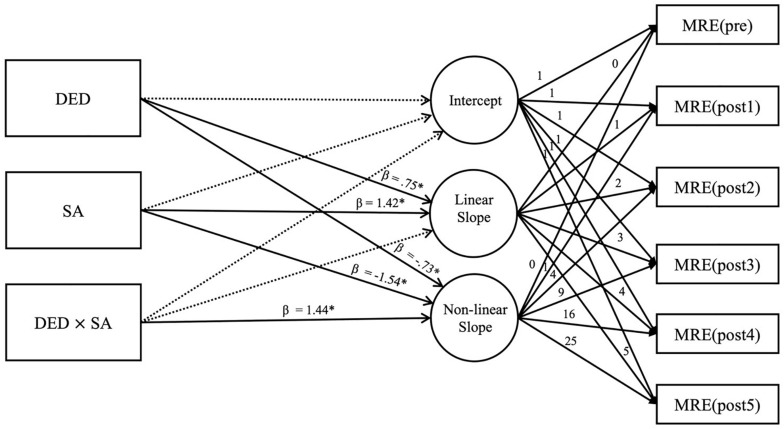
Conditional model. DED, degree of ego depletion; SA, state anxiety; MRE, mean radial error. Linear Slope is the slope of the early-phase trials; Non-linear Slope is the slope of the late-phase trials. Dotted lines are not statistically significant whereas solid lines are. ^∗^*p* < 0.05.

We examined the effects ED dependent upon SA had on the non-linear change in darts performance. As a result, no statistical significance was found in the conditional model shown in Table 2 (χ^2^(21) = 21.42; *p* = 0.434), which suggests that there were no statistically significant differences between the conditional model (shown in Figure 4) and the raw data.

**FIGURE 5:**
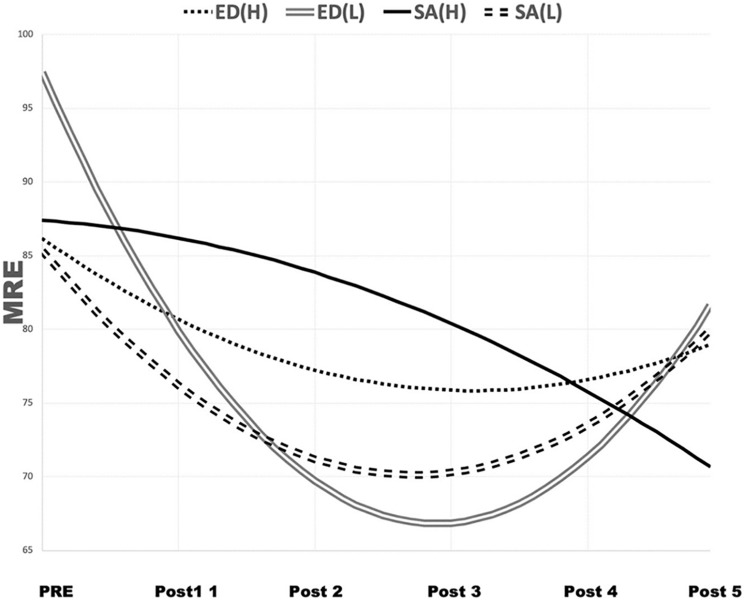
Non-linear change in MRE, reflecting ED and SA. ED, ego depletion; H, above mean; L, below mean; MRE, mean radial error; SA, state anxiety.

The conditional model analysis confirmed the statistically significant positive effects the degree of ED (DED; β = 0.75, *p* < 0.05) and SA (β = 1.42, *p* < 0.05) had on the linear slope. Figure 5 illustrates the non-linear change that occurred in the participants as a result of the effects of DED and SA; in this figure, the participants are divided into below-mean (L) and above-mean (H) groups based on their mean DED and SA scores. As shown in Figure 5, the early-phase dart performance was impaired (i.e., the MRE decreased) as DED and SA increased. This confirms that ED and SA had negative effects on performance improvement in the early-phase trials.

**FIGURE 6:**
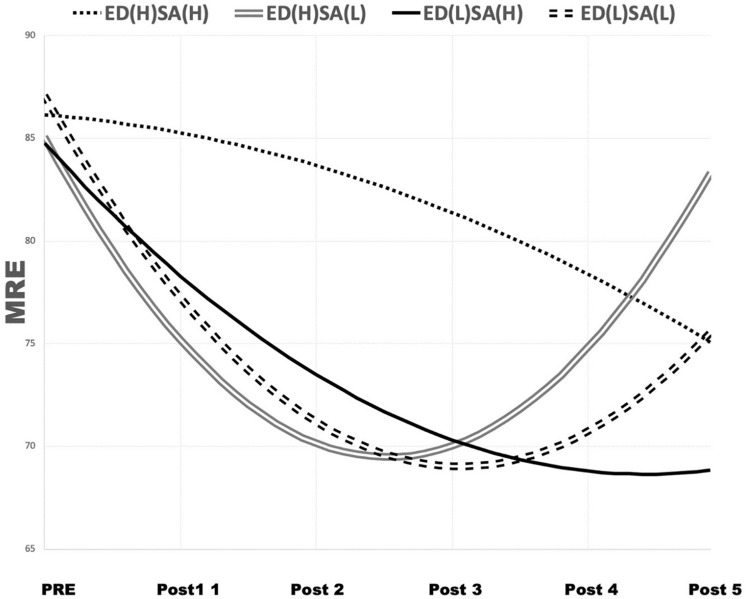
Non-linear change in MRE, reflecting the interaction between ED and SA. ED, ego depletion; H, above mean; L, below mean; MRE, mean radial error; SA, state anxiety.

The interaction between DED and SA (β = 1.44, *p* < 0.05) was found to have a statistically significant positive effect on the non-linear slope. Meanwhile, the slope for late-phase MRE and performance change did not show statistical significance, as shown in Table 2, and this suggests that the effect of DED varies depending on the degree of SA. This phenomenon was observed after dividing the participants into L and H groups based on their mean DED and SA scores, and then into four subgroups: Group 1: ED(H) + SA(H), Group 2: ED(H) + SA(L), Group 3: ED(L) + SA(H), and Group 4: ED(L) + SA(L). Figure 6 illustrates the non-linear change exhibited by each group. This shows that the lower the SA leading to ED, the greater the non-linear slope; that is, in late-phase trials, ED had a negative effect on performance (higher MRE) this tendency was strengthened with lower SA. From these results, it can be inferred that ED had a negative effect on the late-phase performance of the participants with low perceived SA.

## Discussion

Most research studies on SA and motor performance have used analyses of variance (ANOVA; Mullen and Hardy, 2000; Oudejans and Pijpers, 2009; Englert et al., 2015a, b, c). As ANOVAs can be used when the independent variable is a categorical variable and the dependent variable is a continuous one, a typical method of analysis is to distinguish the group whose anxiety is manipulated from the control group, to examine the effects on performance. Therefore, ANOVAs are limited because they do not account for individual differences; they lead researchers to assume that the manipulation of anxiety similarly affects all participants in the group. The SA in this study was measured as a continuous variable using the LCM. Measuring SA as a continuous variable help identify a variety of situations that can affect SA (e.g., worries caused by low dart scores during the experiment) and psychological variables (e.g., emotional discomfort caused by self-exhaustion tasks), which in turn can be considered as individual differences. In an actual sports competition, anxiety is influenced by negative emotions (burnout, depression, etc.) as well as cognitive anxiety due to poor performance, and the degree of anxiety per individual may change even under the same stressful condition. Assigning SA as a continuous variable can be considered as one of the statistical remedies that can reflect actual competitive sports situations.

As shown in Table 2, the MRE of the participants in this study decreased during the early-phase trials, but this decreasing trend was not maintained in the late-phase trials, consequently returning a non-linear relationship. These results are consistent with those of a previous study (Stöckel and Fries, 2013), in which performance changes in basketball players were investigated after the distance for three-point shots in the European professional basketball league was changed from 6.25 to 6.75 m. As a result, the success rate for three-point shots was found to initially increase, only to decrease later as the number of three-point shots taken increased. Although no definite conclusion can be drawn from the results of our study and this previous study, it can be assumed that motor performance is sustained in the form of a quadratic curve (improvement in early-phase trials and deterioration in late-phase trials) as the number of task executions increases.

Another previous study (Englert et al., 2015c), which investigated the relationship between ED and darts performance, reported that ED had a negative effect on concentration, and that this effect was reinforced when participants had a greater state-anxiety level. However, Englert et al. (2015c) did not specify whether the effects of ED, SA, and the interaction between ED and SA appeared in the early or late phases of task executions. The result of our study suggests that ED and SA have a direct effect during the early phases of perceptual-motor task executions, and that the interaction between ED and SA has a direct effect in later phases (producing a non-linear slope). Consequently, the effect of ED on performance may typically occur in the earlier phases of performance. One’s self-control resources can be depleted for a certain amount of time; however, they are also replenished again over time (e.g., Baumeister et al., 1994, 1998). Therefore, performance may have only suffered in the early stages of the dart-throwing because self-control resources were being slowly recovered over time.

In general, in high-anxiety conditions, people tend to concentrate on irrelevant information rather than relevant information (Nieuwenhuys and Oudejans, 2012). For example, people with height phobia perceive heights as being greater than do people without height phobia (Teachman et al., 2008). Further, when judging whether a given stimulus is threatening or non-threatening, anxiety has been found to cause people to devise a more plausible interpretation of the perceived threat (Bishop, 2007; Blanchette and Richards, 2010). In our study, the group with high early-phase SA did not show a decrease in MRE (performance improvement), but the group with low SA did show a decrease in MRE. This finding can be interpreted as showing the negative effect of high SA on performance in the early phases of trials, because participants with high anxiety levels failed to make correct decisions based on darts-related information, which adversely affected their darts performance.

Contrary to our expectations, however, the lowest performance was observed in the group with low SA and high ED. According to the catastrophe model proposed by Hardy (1996), an optimum level of cognitive anxiety helps improve performance, and high cognitive anxiety and physiological arousal lead to an abrupt drop in performance. Englert et al. (2015c) noted that SA-based manipulation of dart-throwing from a 7-m height induces an extreme anxiety situation in participants; this corresponds to a high cognitive anxiety situation in the catastrophe model, and coupling this anxiety situation with ED leads to performance deterioration. In contrast, given the SA (or cognitive anxiety) measured in our study, which occurred in a competition situation and under performance pressure (inter-participant competition and burden regarding consequences of the experiment results), the group with high SA could be considered to have been closer to the optimum level of cognitive anxiety than were the participants in Englert et al.’s (2015c) study, who experienced extremely high SA. This suggests that the optimum level of cognitive anxiety (the high SA group in our study) does not significantly affect performance, even under ED.

The results from this study are helpful in developing an effective cognitive strategy for improving performance. In the early stages of performance, it may be desirable to focus on task-related cues and use cognitive strategies (e.g., keywords, self-talk, etc.) that can help to overcome self-exhaustion and high SA, and in the late performance period to use a strategy (e.g., deep breathing, imagery) that can be used to regulate SA.

Englert (2016) suggested that the effect of ED on performance is overestimated by publication bias, and highlighted the need to solve the lack of consistency in research results by reporting research results indicating that ED does not affect performance (Graham et al., 2014; Englert and Bertrams, 2015). Previous studies that examined the association between ED and sports performance (Dorris et al., 2012; McEwan et al., 2013; Englert and Bertrams, 2014; Wagstaff, 2014; Englert and Wolff, 2015) have reported that the participants in ED-manipulation groups deplete their self-control resource while those in control groups do not. However, Englert and Bertrams (2015) and Graham et al. (2014) argued that participation in ED tasks has no negative effect on performance if autonomy is ensured, and that the relationship between ED and performance can vary depending on the perceived self-efficacy, mood, and motivation (Englert, 2016). Therefore, considering the possible influence individual differences can have on the degree of ED among participants in ED-manipulation tasks, which can depend on the autonomy of participation, perceived self-efficacy in the task, and individual mood and motivation, there is a need for future studies to establish a methodology of reflecting individual differences or completely controlling for all contaminated variables (e.g., autonomy, self-efficacy, mood, and motivation). However, given the practical difficulty in completely controlling for all contaminated variables that may influence dependent variables, reflecting individual differences with regard to the degree of ED appears to be a reasonable approach. In this respect, the consistency of research results among ED-related studies can be ensured by measuring degree of ED as a continuous variable (as in our study); this is because measurements can then be made after considering various individual characteristics and contaminated variables as individual differences.

In the present study, ego-depletion and SA manipulation check were performed via retrospective measurement after the dart-throwing tasks were completed. This experimental design might have the potential to confound dart-throwing tasks to retrospective DED and SA measures. The correlation between DED and MRE, SA and MRE is low (Table 1); therefore, it is unlikely that MRE interferes with the measurement of DED and SA. Due to the nature of the experimental design (i.e., the non-SA [NSA] population does not exist and SA operation confirmation should be performed by recall), the errors due to recall measurements were not completely controlled. In addition, due to the “within subject design,” the first and second “pre-test” are used in the same MRE (pre). There may be concerns that the duplication of MRE (pre) could adversely affect the validity and reliability of the findings. However, as shown in Figure 4, there were no statistically significant effects of DED, SA, or the interaction of DED and SA on the intercept [i.e., MRE (pre)]. The critical dependent variables in this study were the linear and non-linear slope. Therefore, the use of MRE (pre) duplication may have minimal impact on the research results. Nevertheless, further studies must classify the ED/NED and SA/NSA groups using a between-subject design, which would eliminate the errors of recall and the procedural problems associated with MRE (pre) duplication, to confirm the validity of these findings.

Marcora et al. (2009) defined mental fatigue as a psychobiological state caused by prolonged periods of demanding cognitive activity. Hagger et al. (2010, p. 67) in their review article concluded that “mental fatigue is therefore an analog for ED and likely coincides with the depletion of self-control.” On the other hand, Baumeister (2014) showed indicated that fatigue is higher when exposed to self-exhaustion and claimed that exhaustion of self-control resources could induce subjective and psychological fatigue, but research results have been mixed (Englert, 2016). Further study is necessary to distinguish the conceptual differences between psychological fatigue and ED, and examine their effects on exercise performance.

Whereas the effects of ED on performance deterioration have previously been examined in a number of studies, no research has yet investigated whether ED has negative effects on performance in terms of kinematic or dynamic processes. It is possible that various kinematic and dynamic factors may have an influence on different performance outcomes produced as a result of ED-related changes in task-execution movements (e.g., coordination, timing, joint angle, and angular velocity). Further, in this study we examined the aspects of ED, SA, and performance change, but did not investigate the kinematic or dynamic mechanisms associated with performance change. Considering this, a follow-up study that investigates the effects of kinematic or dynamic changes, which may lead to ED, on performance is necessary.

## Data Availability

The datasets generated for this study are available on request to the corresponding author.

## Ethics Statement

The study was conducted in accordance with the Declaration of Helsinki and the protocol was approved by the Ethics Committee which is INUIRB (Incheon National University IRB). All participants provided written informed consent to participate, after being provided with sufficient explanations regarding the purpose, content, and method of the study.

## Author Contributions

JY, KP, and MS conceived the overall study design. JY and MS analyzed the data. All the authors contributed to the manuscript writing.

## Conflict of Interest Statement

The authors declare that the research was conducted in the absence of any commercial or financial relationships that could be construed as a potential conflict of interest.

## Footnotes

1 In general, it is recommended to perform the task within 5 minutes after the end of the ego-depletion task (Englert et al., 2015c). Therefore, if the ego-depletion questionnaire was administered after the ego-depletion manipulation, participants will be recovered from the ego-depletion and affect the experiment results. In addition, as participants may have been able to anticipate the purpose of the study while filling in the manipulation confirmation questionnaires, there is also the possibility that this could distort the results.

2 To measure the dart-throwing task performance, three darts were repeated five times in a batch and were measured a total of 15 times. In other words, the performance was measured by throwing three darts consecutively, removing the darts from the dart board, and then throwing three again. Therefore, the result from each dart-throwing task should be considered as a separate measurement point; the Mean values, a representative value to accommodate each dart result, were used in this study. Moreover, we cannot be certain that any of the three dart tasks performed in this experiment were not affected by the independent variable (i.e., ego-depletion). In this situation, the use of the Median Radial Error, which discards two of the three darts results, cannot represent the experimental results correctly; rather, it can be significantly affected by certain values. Therefore, the authors concluded that the extreme values of the subjects during the three dart tasks were part of the performance and constituted important information.
